# Liver-Kidney Crosstalk in Major Pediatric Diseases: Unraveling the Complexities and Clinical Challenges

**DOI:** 10.3390/jcm14113911

**Published:** 2025-06-02

**Authors:** Dario Piatto, Delia De Biasio, Francesco Giustino Cesaro, Gianmario Forcina, Vittoria Frattolillo, Antonio Colucci, Fabio Lamberti, Pierluigi Marzuillo, Emanuele Miraglia del Giudice, Anna Di Sessa

**Affiliations:** Department of Woman, Child, and General and Specialized Surgery, University of Campania “Luigi Vanvitelli”, 80138 Naples, Italy; dario.piatto@studenti.unicampania.it (D.P.); delia.debiasio@studenti.unicampania.it (D.D.B.); francescogiustino.cesaro@studenti.unicampania.it (F.G.C.); gianmario.forcina@studenti.unicampania.it (G.F.); vittoria.frattolillo@studenti.unicampania.it (V.F.); antonio.colucci1@studenti.unicampania.it (A.C.); fabio.lamberti@studenti.unicampania.it (F.L.); pierluigi.marzuillo@unicampania.it (P.M.); emanuele.miraglia@unicampania.it (E.M.d.G.)

**Keywords:** liver, kidney, crosstalk, metabolic dysfunction-associated steatotic liver disease, hepatorenal syndrome, pediatric diseases, management, challenges

## Abstract

The liver and kidneys are two of the most vital organs, each with distinct but overlapping functions essential for maintaining homeostasis. The complex interplay between these organs, commonly referred to as liver-kidney crosstalk, plays a crucial role in the pathophysiology of several acute and chronic conditions in childhood. Despite its importance, the precise biological mechanisms driving this interaction remain incompletely understood. This crosstalk is particularly significant in various pediatric diseases (e.g., Metabolic Dysfunction-Associated Steatotic Liver Disease (MASLD), Hepatorenal Syndrome (HRS), genetic and metabolic disorders, etc.) where shared pathophysiological factors—including systemic inflammation, metabolic disturbances, oxidative stress, and vascular dysfunction—simultaneously affect both organs. Clinically, this interaction presents unique challenges in diagnosing, managing, and treating liver-kidney diseases in affected children. Understanding the pathogenic mechanisms underlying liver-kidney crosstalk is essential for improving patient care and outcomes through an integrated, multidisciplinary approach and personalized treatment strategies. This review aims to explore liver-kidney crosstalk in key pediatric diseases, offering a comprehensive overview of current knowledge, clinical challenges, and potential therapeutic interventions in this complex field.

## 1. Introduction

Liver and kidney diseases are closely interrelated due to their shared roles in metabolic and circulatory functions, both of which are critical for maintaining overall health [1]. These organs are vital for processes such as detoxification, metabolism, fluid balance, and hormonal regulation [1]. Dysfunction in one organ often leads to impairment in the other, presenting significant challenges for diagnosis, management, and prognosis [1,2,3].

Both liver and kidney diseases share several common risk factors, such as metabolic dysfunction and chronic inflammation [4,5]. Notably, chronic inflammation may play a crucial role in the progression of damage to both organs [2,4,5].

Clinically, this interrelationship manifests in various pediatric conditions, ranging from well-defined diseases such as hepatorenal syndrome (HRS), polycystic diseases, and metabolic-associated steatotic liver disease (MASLD), to disorders like nephrotic syndrome, glomerulopathies, tubulopathies, acute kidney injury (AKI), and certain genetic and metabolic diseases (e.g., Alport syndrome, Fabry disease, Wilson disease, etc.) [6,7].

Although HRS is more commonly observed in adults with advanced liver disease, particularly cirrhosis [8], it can also occur, though rarely, in children [9]. In this age group, this life-threatening condition may result from acute liver failure, fulminant hepatitis, biliary atresia, or chronic liver disease, such as cirrhosis and liver failure [9]. Pathogenic mechanisms underlying HRS involve impaired hepatic function in toxin processing and blood circulation in advanced liver disease [9].

The pathophysiological interplay between liver and kidney diseases becomes increasingly evident in children with MASLD, as shared metabolic abnormalities, such as insulin resistance (IR) and inflammation, predispose to kidney damage (KD) development [2].

Given the close relationship between liver and kidney dysfunction, an integrated approach to management is needed, with treatments addressing both organ systems [2,4]. A deeper understanding of the interplay between these organs is essential for improving outcomes in pediatric patients [5,6,7].

We aimed to provide a comprehensive overview of the key clinical manifestations of the multifaceted liver-kidney interconnection in the pediatric population.

## 2. Hepatorenal Syndrome

HRS is a severe complication of liver cirrhosis, resulting from the interplay of several pathogenic mechanisms [8,9].

Portal hypertension is a key pathophysiological feature, primarily resulting from increased hepatic resistance due to liver nodularity and the activation of hepatic stellate (Ito) cells, which leads to sinusoidal narrowing [8].

The resulting increase in intrahepatic resistance stimulates the release of nitric oxide from the splanchnic vasculature, promoting vasodilation and further increasing portal blood flow [10,11,12]. Blood sequestration in the splanchnic circulation creates hypovolemia, systemic hypotension, and renal hypoperfusion, activating compensatory vasoconstrictor mechanisms that worsen kidney dysfunction [10,13].

Inflammation also plays a key role in HRS. Increased splanchnic pressure causes intestinal congestion and bacterial translocation, activating Kupffer cells and releasing pro-inflammatory cytokines [13,14]. This cascade promotes hepatic stellate cell activation and intrahepatic fibrosis [14,15]. Elevated levels of cytokines like interleukin-6, tumor necrosis factor (TNF)-alpha, and monocyte chemoattractant protein-1 are significantly higher in cirrhotic patients with AKI compared to both healthy controls and cirrhotic patients without AKI [13,15].

Although rare, pediatric HRS has been documented [9,16,17,18,19], with an incidence of approximately 5% in children with chronic liver diseases prior to liver transplantation [9].

Several mechanisms contribute to its development, including marked splanchnic vasodilation, release of vasoactive mediators, a hyperdynamic circulatory state, and the onset of cardiac dysfunction [9]. Additionally, neurohormonal alterations, including dysregulation of the sympathetic nervous system and the renin-angiotensin system, as well as the involvement of vasopressin, play significant roles in the pathophysiology of pediatric HRS [9,10].

HRS in childhood is a life-threatening complication associated with advanced liver cirrhosis (e.g., biliary atresia, autoimmune hepatitis, metabolic liver disorders, portal hypertension), acute liver failure (e.g., viral infections, sepsis, drug-induced liver injury), liver transplantation, sepsis, or infections, and is characterized by kidney failure without intrinsic kidney disease [9,10,14].

Two forms of pediatric HRS have been identified such as Type 1, an acute and rapidly progressive form often triggered by factors such as gastrointestinal bleeding or spontaneous bacterial peritonitis, and Type 2, a more slowly progressive form of kidney failure that typically develops in the context of chronic ascites [9,14].

Diagnosis of pediatric HRS is based on clinical criteria, including the absence of significant proteinuria or structural kidney disease [9]. Management primarily targets the underlying liver dysfunction, with liver transplantation as the definitive treatment, along with supportive measures such as kidney transplant when needed [9,10]. If left unaddressed, HRS is associated with significant morbidity and poor long-term outcomes [9,19]. In addition to timely diagnosis and careful management, future research should focus on developing early biomarkers and alternative treatments to mitigate complications and improve the long-term health of these at-risk patients [9,10].

## 3. Genetic and Metabolic Disorders

Several major pediatric genetic disorders reflect the critical functional crosstalk between the liver and kidneys [6,7].

Although Alport syndrome—a hereditary disorder caused by mutations in type IV collagen genes—primarily affects the kidneys, ears, and eyes, recent findings suggest an intriguing interplay between the liver and kidneys that may modulate disease progression and systemic consequences. This connection is largely attributed to shared inflammatory, metabolic, and fibrotic pathways [6,7].

Fabry disease, an X-linked lysosomal storage disorder caused by mutations in the α-galactosidase A (GLA) gene, leads to the accumulation of globotriaosylceramide, resulting in progressive kidney impairment and potentially affecting hepatic function as well [6,7].

Moreover, crosstalk between the liver and kidneys plays a critical role in the pathophysiology of other rare pediatric genetic disorders [6,7]. For instance, glycogen storage diseases (e.g., types I and III) and cystinosis are both characterized by hepatomegaly and renal tubular dysfunction [6,7]. Wilson disease, a rare autosomal recessive disorder caused by mutations in the ATPase Copper Transporting Beta (ATP7B) gene, leads to impaired hepatic copper excretion. This results in progressive copper accumulation, primarily in the liver, but also in extrahepatic tissues including the kidneys, causing tubular dysfunction, aminoaciduria, and nephrolithiasis [6,7].

Therefore, understanding liver–kidney interactions in pediatric genetic diseases is crucial for early diagnosis and comprehensive clinical care aimed at preserving renal function and improving long-term outcomes.

## 4. Metabolic Dysfunction-Associated Steatotic Liver Disease

Over the past three decades, the prevalence of non-alcoholic fatty liver disease (NAFLD) has significantly increased, affecting about 38% of the global adult population and 13% of children, with rates rising to 34.2% among those with obesity [20,21]. The nomenclature of this liver disease has evolved to better reflect its metabolic origins [22,23,24]. Initially defined by the exclusion of other liver diseases, NAFLD was criticized for its lack of specificity [21,22]. In 2020, the term metabolic dysfunction-associated fatty liver disease (MAFLD) was introduced to focus on metabolic dysfunction as the primary cause [22,23]. In 2023, the term was further refined to metabolic dysfunction-associated steatotic liver disease (MASLD) to improve diagnostic accuracy, prevention, and treatment strategies [24]. However, the clinical and prognostic accuracy of MASLD compared to NAFLD and MAFLD remains debated [25,26].

With rising rates of pediatric obesity and type 2 diabetes (T2D) globally, MASLD has emerged as a prevalent and serious cardiometabolic disease in children [27,28,29]. Epidemiological studies indicate that MASLD is an independent risk factor for cardiovascular diseases (CVD), especially in subtypes such as MASLD, metabolic-associated liver disease (MetALD), and alcohol-associated liver disease (ALD), which are linked to higher fibrosis risk [28,30,31].

A complex inter-organ communication, driven by metabolic dysfunction such as IR and visceral fat accumulation, plays a crucial role in linking MASLD to various extrahepatic conditions, including cardiovascular disease (CVD), thyroid dysfunction, chronic kidney disease (CKD), and malignancies [28,32,33,34]. In particular, CKD represents a well-established risk factor for end-stage kidney disease (ESKD) and CVD [35]. Growing evidence suggests shared pathogenic mechanisms between MASLD and CKD, including IR, inflammation, and oxidative stress [4,36].

While most evidence linking MASLD to KD is currently available in adults [37,38,39,40], emerging studies have also highlighted this association in children [41,42,43]. Notably, KD in children may be heterogeneous [44], with glomerular hyperfiltration serving as an early marker of kidney injury in conditions such as diabetes, hypertension, and obesity [41].

In addition to CKD, MASLD may progress to fibrosis and cirrhosis more rapidly in adults than in children, with an increased risk of hepatocellular carcinoma (HCC) [45,46]. Furthermore, growing evidence suggests an elevated risk of extrahepatic cancers related to MASLD, including thyroid cancer, female genital tract cancers, and urinary tract and gastrointestinal cancers [28,46].

Given the combined burden of both diseases [4,29,30,33], a deeper understanding of the complex pathophysiological mechanisms underlying MASLD and CKD is essential to improve the overall management of these at-risk patients.

## 5. Potential Mechanisms Linking MASLD to CKD

**Figure 1:**
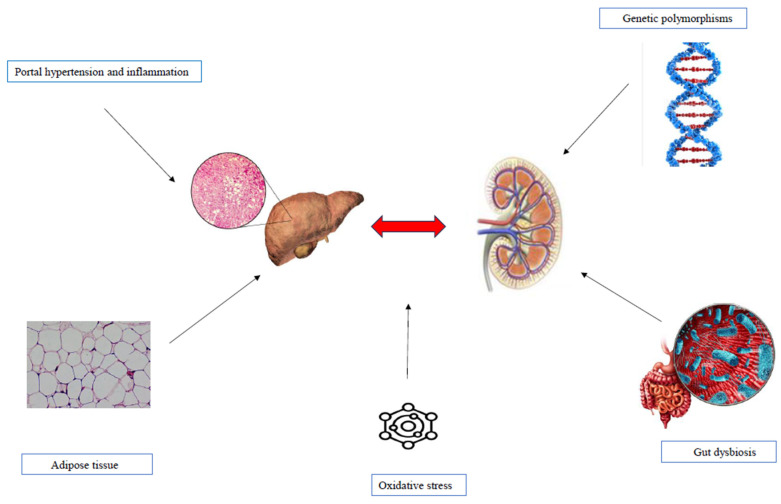
Pathophysiological mechanisms underlying MASLD and KD.

The pathogenetic mechanisms linking MASLD and CKD remain an area of ongoing research, as they are not yet fully understood [4,32,33,34]. However, they appear to involve a shared set of cardiometabolic risk factors, including oxidative stress, IR, and inflammation. These mechanisms are characterized by proinflammatory and profibrotic molecular patterns, as well as the involvement of adipose tissue, lipid droplets, and Peroxisome proliferator-activated receptor-gamma (PPAR-γ) [32,33,34]. Additionally, intestinal dysfunction and dysbiosis, along with genetic and epigenetic polymorphisms, have been identified as contributing factors in the pathogenesis [4,47,48,49,50] (Figure 1).

### 5.1. IR and Dysmetabolism

Evidence from both adult and pediatric studies consistently identifies IR as a key factor linking kidney and liver dysfunction [48,51,52,53]. Several cardiometabolic features of MASLD contribute not only to CVD but, more significantly, to CKD, mainly through the promotion of low-grade inflammation and vascular alterations [4]. Additionally, pro-atherogenic dyslipidemia may increase CKD risk by altering plasma lipoprotein concentrations and the composition of small molecules, proteins, and fatty acids [54].

Renal lipid accumulation, also known as “Fatty Kidney”, plays a crucial role in linking MASLD and CKD, particularly in children with obesity who develop CKD [4,54]. Lipid accumulation in renal podocytes induces IR, causing structural damage to glomeruli and tubules. Lipid droplets in the perirenal space, kidney sinus, and parenchyma each exert distinct effects on renal structure and function [4]. In the context of MASLD, early disturbances in portal and splanchnic vasoregulation can activate a pathological “hepatorenal reflex”, potentially preceding cirrhosis and HRS [55]. This reflex is driven by elevated intrahepatic vascular resistance and altered sinusoidal blood flow [56]. Subclinical portal hypertension may further promote hepatic inflammation and fibrosis, contributing to renal dysfunction and CKD progression [55].

Altered hepatokine release in MASLD may contribute to CKD through liver-kidney crosstalk [4,57,58]. Fibroblast growth factor-21 (FGF-21), a key hepatokine, has garnered attention due to its receptor agonist, efruxifermin, which has shown promise in phase 2 trials for SLD [4]. Elevated FGF-21 levels are observed in conditions such as T2D, CKD, and SLD [59,60,61], reflecting an adaptive response to hyperglycemia and IR [62]. However, in chronic conditions such as CKD and MASLD, resistance to FGF-21 may attenuate its beneficial effects [60]. Despite its effectiveness in improving T2D and IR markers, the direct impact of FGF-21 on kidney function remains to be fully elucidated [60].

### 5.2. Genetics

Genetic factors play a crucial role in the pathogenesis of both MASLD and CKD, with shared genetic variants contributing to liver and kidney dysfunction, primarily through mechanisms involving inflammation, fibrosis, lipid metabolism, and oxidative stress [50,63]. Strong evidence highlights the involvement of polymorphisms in genes such as Patatin-like phospholipase domain-containing protein 3 (*PNPLA3*), Transmembrane 6 superfamily member 2 (*TM6SF2*), Hydroxysteroid 17-beta dehydrogenase 13 (*HSD17B13*), and Membrane-bound O-acyltransferase domain-containing 7 (*MBOAT7*) in the development and progression of both diseases [50,64].

Among these, the *PNPLA3* gene polymorphism rs738409 C>G represents the most robust determinant of hepatic steatosis susceptibility influencing lipid metabolism through the hydrolysis of triglycerides and the regulation of fat storage within the liver [65,66,67].

Recent estimates suggest that approximately 15% of the European population with MASLD carries this risk allele, underscoring its potential as an early biomarker for MASLD identification [68,69].

Beyond its primary expression in hepatic stellate cells, this risk polymorphism is also highly expressed in the kidney, where it promotes lipid accumulation [1,55,68]. The resulting lipid buildup increases oxidative stress, triggers proinflammatory cytokine production, and induces IR [68]. Consequently, mesangial cells transform into foam cells, impairing contractility, while podocyte apoptosis contributes to glomerulosclerosis, reduced eGFR, and proteinuria in both adults and children [1,55]. Emerging evidence links this polymorphism not only to hepatic steatosis but also to KD, highlighting its broader role in metabolic dysfunction [50,63].

Both adult and pediatric patients carrying the I148M polymorphism of the *PNPLA3* gene exhibit reduced eGFR and elevated alanine transaminase levels compared to noncarriers [67,68]. This polymorphism negatively impacts eGFR, particularly in adults with metabolic dysfunction, increasing the risk of tubular and glomerular damage regardless of the severity of hepatic steatosis [70,71,72,73]. Notably, the *PNPLA3* rs738409 polymorphism also affects kidney function in conditions unrelated to metabolic dysfunction and MASLD, such as chronic HCV infection and CKD, even in the absence of traditional risk factors for kidney disease [74].

Pediatric studies support the detrimental effect of the I148M polymorphism on renal function, even in children with obesity and prediabetes [75,76,77]. This was further confirmed in a large Italian study assessing kidney function in 1037 children with obesity and MASLD [42].

Noteworthy, the *PNPLA3* rs738409 polymorphism has been found to be more common in individuals with hepatic steatosis, even in the absence of obesity and metabolic dysfunction [78,79]. However, recent evidence also suggests that IR plays a pathogenic role in MASLD development independently of the presence of this genetic variant [80].

More recently, a notable relationship between genetic background and the intestinal microbiome in MASLD development has emerged [81,82]. Specifically, individuals with the PNPLA3 rs738409 CC and CG genotypes demonstrated a reduction in intestinal Blautia and Ruminococcaceae, potentially amplifying the role of microbiota in the onset and progression of MASLD [81]. This may facilitate the development of targeted therapeutic strategies [81].

In addition to *PNPLA3* gene, the E167K polymorphism (rs58542926) of the *TM6SF2* gene has been also implied in MASLD development and progression both in adults and children [50,63,83,84]. Although its exact function remains to be elucidated, this gene has been demonstrated to be involved in lipid metabolism and transport [83], with a strong correlation with MASLD development [74,83,84]. Emerging evidence suggests that this risk polymorphism also exerts a role in KD development, through mechanisms involving lipid accumulation, oxidative stress, and inflammation, ultimately promoting fibrosis and impairing kidney function [50,74,85,86].

In this complex landscape, the *MBOAT7* rs641738 polymorphism has been identified as playing a pathophysiological role across all age groups, contributing to both MASLD and KD development [63,85]. This variant affects lipid metabolism and inflammation pathways, which are key drivers in the progression of both liver and kidney diseases [63,85]. Specifically, MBOAT7 has been implicated in lipid accumulation, a process that leads to the development of fibrosis [87].

Another gene implicated in hepatic steatosis is *HSD17B13*, although its functional role remains incompletely understood [87,88,89]. This variant appears to have a protective effect against hepatic inflammation and fibrosis progression, as well as KD associated with MASLD [50,88]. However, overexpression of HSD17B13 has been observed in fibrotic hepatic tissue from patients with MASLD [89]. An insightful study used the *PNPLA3*, *TM6SF2*, and *HSD17B13* polymorphisms as part of a genetic risk score to diagnose significant fibrosis in MASLD, though no statistically significant results were found [66].

Overall, genetics represents a key determinant in the development and progression of both MASLD and CKD [55,68,87], but further research is needed to fully understand its complex interaction with environmental factors in the pathophysiology of both diseases.

### 5.3. Oxidative Stress

Evidence from preclinical models indicates that oxidative stress may be a key driver in the development of both MASLD and CKD [90,91,92].

Oxidative stress, resulting from an imbalance between oxidants and antioxidants, triggers lipotoxicity, lipid peroxidation, endoplasmic reticulum stress, and mitochondrial dysfunction, driving the pathogenesis of MASLD [93]. Nuclear factor erythroid 2-related factor 2 (Nrf2), a key transcription factor, regulates these processes by inhibiting genes linked to hepatic fat accumulation and neutralizing reactive oxygen species (ROS), thereby protecting hepatocyte mitochondrial function and cellular integrity [94].

Induced by Nrf2, heme oxygenase-1 (HO-1) defends against oxidative damage, inflammation, and apoptosis, thus preventing MASLD progression [95]. Oxidative stress activates Nrf2, which is released from Keap1 to induce protective genes. Dysregulation of Nrf2 and excess ROS are influenced by fetuin-A and Klotho [95,96]. Fetuin-A, a liver-derived glycoprotein, reduces adiponectin levels, worsening metabolic disorders, while Klotho, an antioxidative protein, modulates ROS. Reduced Klotho expression under oxidative stress enhances inflammation and thrombosis. Additionally, indole sulfate, regulated by Klotho, contributes to platelet activation, linking oxidative stress to CKD risk [1,96].

In MASLD, oxidative stress mediates hepatocellular damage, inflammation, and the progression from simple steatosis to advanced stages such as metabolic dysfunction-associated steatohepatitis (MASH), disrupting cellular integrity, inducing fibrosis, and accelerating cirrhosis [90,91]. Similarly, in CKD, oxidative stress causes kidney cell injury, exacerbates inflammation, and promotes interstitial fibrosis, driving the decline in renal function and progression to ESKD [90,91]. In both conditions, oxidative stress is a key driver of pathophysiology, highlighting its potential as a therapeutic target [4,90,91].

### 5.4. Lipid Metabolism

Lipid metabolism contributes to both MASLD and CKD, with interrelated mechanisms involving IR, inflammation, dyslipidemia, and oxidative stress [4,47,97]. Dysregulation of lipid handling in the liver and kidneys accelerates disease progression and contributes to complications, particularly CVD [4,98].

In MASLD, impaired fatty acid oxidation and dyslipidemia lead to hepatic fat accumulation, triggering inflammation and fibrosis [97]. Reduced fatty acid oxidation results in triglyceride buildup and hepatocellular steatosis, while elevated free fatty acids from adipose tissue or diet overwhelm the hepatic processing capacity, promoting lipid deposition, inflammation, and oxidative stress [97]. This cascade contributes to hepatic injury and fibrosis. IR further increases Very Low-Density Lipoprotein (VLDL) production, raising circulating triglycerides and cholesterol, which worsens fat accumulation. Additionally, dysregulated adipokine secretion from adipose tissue exacerbates chronic inflammation, intensifying IR and liver damage [97,98].

In CKD, disrupted lipid metabolism leads to elevated atherogenic lipids, increasing cardiovascular risk and contributing to KD [98,99]. Impaired kidney function hinders lipoprotein clearance, causing the buildup of atherogenic lipids such as Low-Density Lipoprotein (LDL) and VLDL, which exacerbate cardiovascular risk [99]. Uremic toxins induce oxidative stress, triggering lipid peroxidation, which worsens inflammation and endothelial dysfunction. Additionally, impaired fatty acid metabolism promotes lipid accumulation in various tissues, including the kidneys, leading to kidney lipotoxicity, fibrosis, and progressive KD [99]. These interconnected mechanisms accelerate CKD progression and its associated cardiovascular complications [4,98,99].

However, the relationship between dyslipidemia and kidney outcomes in CKD remains debated [4,100]. While LDL cholesterol levels are linked to mortality and hospitalizations due to CVD, they are also influenced by CKD progression [100]. Low HDL cholesterol and impaired HDL functionality are associated with worse prognosis and CKD progression [101]. Reduced lecithin-cholesterol acyltransferase (LCAT) levels also predict CKD progression, regardless of pre-existing kidney dysfunction [102]. In atherogenic dyslipidemia, elevated Sterol Regulatory Element-Binding Protein (SREBP) promotes kidney lipotoxicity and fibrosis by activating TGF-β, accelerating CKD progression [103,104].

Considering the complex interplay of lipid metabolism with IR and inflammation in the development and progression of MASLD and CKD [4,47], managing lipid levels is crucial for slowing the progression of both diseases.

### 5.5. Fructose Metabolism

A high-fructose diet significantly contributes to the pathogenesis and progression of both MASLD and CKD [4,105,106]. Mechanisms include increased pro-inflammatory cytokine production, endothelial dysfunction, mitochondrial oxidative stress from ROS, nitric oxide depletion impairing vascular homeostasis, and reduced hepatic adenosine triphosphate (ATP), all promoting de novo lipogenesis in liver and kidney tissues [4].

Fructose metabolism is central to MASLD pathogenesis, enhancing hepatic lipogenesis, oxidative stress, and inflammation [105,106]. Unlike glucose, fructose is rapidly phosphorylated in the liver by fructokinase (KHK), bypassing phosphofructokinase-1 (PFK-1) regulation, leading to unregulated lipogenic flux, triglyceride accumulation, hepatic IR, and increased uric acid production, which exacerbates oxidative stress and mitochondrial dysfunction [4,107]. Uric acid not only links to cardiometabolic diseases [107,108], but also activates enzymes in the polyol pathway, increasing endogenous fructose biosynthesis, worsening triglyceride accumulation, and accelerating steatosis progression [1].

A randomized controlled trial by Schwimmer et al. found that reducing free sugar intake significantly lowered hepatic fat content in adolescents with MASLD, supporting fructose restriction as a therapeutic strategy [109].

Excessive fructose consumption also contributes to the pathogenesis and progression of CKD by promoting inflammation, oxidative stress, and the accumulation of nephrotoxic metabolites in the kidneys, exacerbating glomerular injury and accelerating kidney dysfunction [110]. These effects not only worsen CKD in individuals with pre-existing kidney impairment but also increase susceptibility to CKD in healthy individuals [110].

Additionally, fructose disrupts gut microbiota balance, increases intestinal permeability, and enhances endotoxemia, further amplifying hepatic inflammation [110].

Based on these premises, targeting fructose metabolism through dietary interventions or enzyme-specific inhibitors could be a promising strategy for MASLD prevention and treatment [111,112].

### 5.6. Adipose Tissue and PPAR-γ Dysfunction

The role of adipose tissue dysfunction and PPAR-γ signaling has been well-documented in MASLD and CKD pathophysiology [4,113].

Impaired adipose tissue function, especially in obesity, leads to IR, inflammation, and disrupted lipid metabolism, key factors in metabolic diseases [4]. Perirenal fat accumulation is linked to CKD, impairing kidney function via bioactive molecules like adipokines, pro-inflammatory mediators, and reactive oxygen species [4,113,114,115]. This fat buildup contributes to kidney inflammation, fibrosis, and hypertension, accelerating CKD progression [4]. Additionally, lipid deposition in the renal parenchyma, especially in the cortex and medulla, is associated with glomerulosclerosis and proteinuria [113]. PPAR-γ, a regulator of adipocyte function, lipid storage, and metabolic homeostasis, is crucial in managing ectopic fat in the liver and kidneys [4,116]. Disruption of PPAR-γ signaling impairs adipose tissue function, promoting IR, lipid metabolism dysregulation, and fibrosis, thus accelerating both MASLD and CKD progression [4,117,118,119].

These processes highlight the importance of adipose tissue and PPAR-γ as potential therapeutic targets for MASLD and CKD management [4,119].

### 5.7. Gut Dysbiosis

Gut dysbiosis has been implicated in the pathogenesis of both MASLD and CKD through several interconnected pathophysiological mechanisms [4,110,120,121,122]. It disrupts the intestinal barrier, promoting increased permeability (“leaky gut”) and endotoxemia, which leads to systemic inflammation [123]. Dysbiosis also alters microbial metabolism, elevating the production of pro-inflammatory metabolites, such as lipopolysaccharides and uremic toxins, further exacerbating liver and kidney damage [124,125]. Additionally, microbial imbalances affect bile acid and short-chain fatty acid profiles, contributing to metabolic dysfunction and accelerating disease progression via the gut-liver and gut-kidney axes [124,126,127].

A deeper understanding of the intricate relationship between gut microbiota and these chronic diseases offers promising therapeutic avenues aimed at restoring microbial balance and mitigating disease progression [123].

## 6. Evidence on MASLD and KD in Childhood

The relationship of MASLD with KD has been widely demonstrated in adults [4,128,129,130], while similar evidence in childhood is still limited [42,131].

Emerging evidence strongly supports an association between MASLD and an increased risk of developing CKD, particularly in adults [4,132,133]. A large-scale 10-year follow-up study of 12,138 adults with SLD revealed that 1963 participants (16.2%) developed new-onset CKD, with an overall incidence rate of 23.0 per 1000 person-years (24.4 for men and 20.3 for women) [128]. This study confirmed a significant independent association between MASLD and CKD, with this relationship remaining robust even after adjusting for common cardiometabolic risk factors [128].

A large observational cohort study explored the relationship of fibrosis with CKD risk and differences in mortality risk in 2036 adult subjects with MASLD [129]. Authors demonstrated that liver fibrosis was an independent risk factor for CKD in patients with MASLD. An increased mortality risk in patients with fibrosis than those without fibrosis [129] was also reported, further amplified in subjects having both fibrosis and CKD [129].

Despite this robust evidence in adults, data supporting MASLD to KD in children are emerging [28,43,131] (Table 1).

Over the past decades, a large-scale cohort study of Vivante et al. examined the relationship between body mass index (BMI) in 1.2 million adolescents and the risk of developing ESKD later in life [134]. Authors demonstrated a strong dose-dependent association between higher BMI and increased risk for ESKD in adulthood, even in the absence of diabetes or hypertension [134]. This underscores the crucial role of preventing and managing pediatric obesity to reduce the risk of kidney disease later in life [44,135,136].

Given the pivotal role of obesity and dysmetabolism in both kidney damage and MASLD development, the interplay among liver and kidney function has recently garnered scientific attention for its broader impact on overall cardiometabolic health [4,135]. A large study on 234,488 participants found that the presence of common cardiometabolic risk factors such as hypertension (OR 1.35, 95% CI 1.35–1.72), T2D (OR 1.89, 95% CI 1.06–3.38), the number of metabolic syndrome (MetS) traits (OR 1.94, 95% CI 1.75–2.15), and liver fibrosis (OR 4.29, 95% CI 3.36–5.47) significantly increased the risk of prevalent CKD in MASLD context. Of note, over 13.6 years median follow-up, MetS was associated with an increased risk of developing ESKD (HR 1.70, 95% CI 1.19–2.43) [133].

In line with adult findings [132] and the shift in nomenclature from NAFLD to MASLD [24], research has also begun exploring the relationship between the risk of KD and MASLD in childhood [131,137,138]. Although the challenge of the heterogeneous definition of kidney impairment in childhood [43,44], evidence in the field showed an increased risk of developing KD in children with MASLD, particularly in the context of pediatric obesity [44,78,131]. Indeed, the shared interconnected metabolic pathways influencing both liver and kidneys further enhance the dymetabolic environment intrinsically observed in children with obesity, leading to a vicious cycle amplifying organ dysfunction [44,137,138]. However, the optimal pediatric label for identifying SLD and its subsequent impact on cardiometabolic long-term outcomes in children is still debated [139].

In this perspective, additional research is needed to thoroughly explore the relationship between MASLD and the risk of KD development in childhood, particularly considering the consequences of its complex pathophysiological interplay with metabolic dysfunction later in life [4,133].

## 7. New Treatment Perspectives for MASLD

In light of the growing cardiometabolic burden of MASLD on pediatric health and its significant financial impact on healthcare systems [140,141], optimizing its management—encompassing early identification of at-risk patients, treatment, and preventive strategies—is of paramount importance [141,142,143].

Despite substantial evidence supporting the therapeutic role of nutritional and lifestyle interventions [142,143,144,145], no approved pharmacological treatments are currently available for children with MASLD [142]. Pediatric clinical trials assessing the efficacy of metformin, vitamin E, probiotics, and polyunsaturated fatty acids have yielded limited evidence [142,144]. Consequently, lifestyle changes—including diet, physical exercise, and behavioral interventions—remain the cornerstone of MASLD management in childhood [143,144,145,146]. In line with findings in adults [147,148], bariatric surgery has also been proposed as a potential treatment to improve MASH and liver fibrosis in adolescents, though evidence remains limited [146].

**Figure 2:**
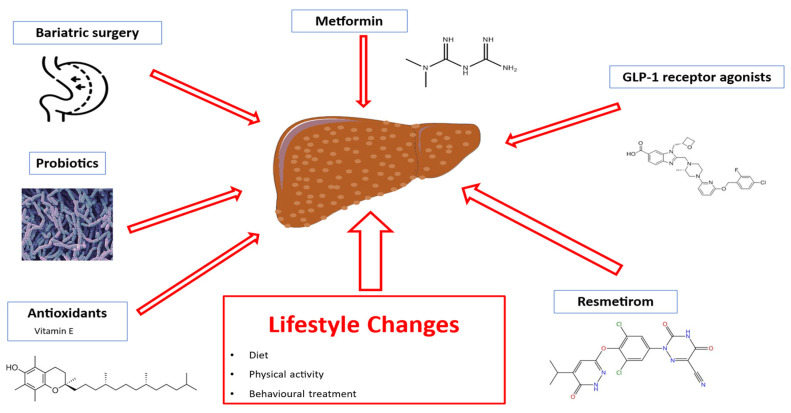
Therapeutic options for MASLD treatment. Abbreviations: GLP-1, glucagon-like peptide 1.

The recent conditional FDA approval of Resmetirom, an orally administered, liver-targeted thyroid hormone receptor (THR)-β selective drug, for treating adults with non-cirrhotic MASH and moderate to advanced fibrosis, marks a significant advancement in MASLD management [149]. This drug, based on the established link between thyroid function and MASLD [150,151,152], has shown promise in reducing hepatic fibrosis, inflammation, and liver lipid content in a large phase III trial, with a favorable safety profile [149]. However, no pediatric studies are currently planned. Preliminary findings on weight-reducing agents like GLP-1 receptor agonists indicate potential for treating pediatric MASLD [153,154] (Figure 2).

Additionally, emerging insights from omics-based research hold potential for advancing treatment strategies [155,156].

Although treatment in childhood remains primarily focused on lifestyle interventions [143,146], pediatric studies are crucial for developing safe and effective treatment options, particularly given the increasing prevalence of MASLD in children and its strong association with cardiometabolic comorbidities and long-term adverse health outcomes [46,146,157].

## 8. Clinical Implications for the Practical Management of KD

Early identification and management of KD in pediatric patients are essential to prevent long-term complications such as CKD and its associated comorbidities. Clinical awareness of liver–kidney crosstalk is increasingly important, particularly in systemic conditions such as sepsis, acute liver failure, genetic and metabolic disorders, HRS, and MASLD, where KD may be subtle or secondary.

Practical management should emphasize early monitoring of kidney function, cautious use of nephrotoxic agents, and timely initiation of supportive therapies, including kidney replacement therapy when indicated.

Given the increasing recognition of MASLD as multisystem disease and its close relationship with obesity [4,28,29], this is particularly concerning in the context of pediatric obesity.

Indeed, children with obesity are at increased risk for early KD, which may progress to CKD and contribute to a broader cardiometabolic burden [44,137,138]. Shared risk factors—including IR, inflammation, and dyslipidemia—warrant proactive and individualized management strategies. In this regard, recent national clinical guidelines recommend early screening starting at six years of age, including serum creatinine and microalbuminuria assessments [135]. Moreover, practical management involves risk-based follow-up according to family history of cardiometabolic disease, presence of hepatic steatosis, and features of metabolic dysfunction [44].

Children classified as low-risk should undergo annual monitoring of blood pressure and albuminuria, with serum creatinine testing every 24 months for prepubertal children and annually for pubertal children [44]. Intermediate-risk children require annual monitoring of blood pressure, albuminuria, and biochemical evaluations, while high-risk children need more frequent monitoring—blood pressure and albuminuria every three months, with biochemical testing every six months [44].

Prompt referral to a pediatric nephrologist in the presence of KD is of paramount importance. Early lifestyle interventions, regular kidney function monitoring, and tailored follow-up care are key to preventing long-term kidney and cardiometabolic complications in this population [44,137,138].

Overall, a multidisciplinary approach involving pediatric nephrologists, hepatologists, and critical care specialists is crucial for optimizing patient outcomes.

## 9. Conclusions

Liver and kidney diseases in childhood are closely linked, sharing common pathophysiological mechanisms and risk factors such as metabolic dysfunction, IR, oxidative stress, and chronic inflammation [4,137]. Early diagnosis and a comprehensive, integrated management approach are critical to addressing both hepatic and kidney dysfunction in affected children [2,4,7].

Further research is needed to better understand the molecular pathways driving these conditions, identify biomarkers for early detection, and develop targeted therapies. A multidisciplinary approach is essential to optimize care and improve outcomes for children with these interconnected diseases.

## 10. Future Directions

As the understanding of the liver-kidney axis in pediatric populations continues to evolve [5,7], future research should focus on elucidating the molecular mechanisms linking these organs. Key areas of interest include metabolic dysfunction, insulin, IR, inflammation, and oxidative stress, which drive the progression of both liver and kidney diseases. Identifying early biomarkers for these conditions is crucial for improving diagnostic accuracy and enabling timely interventions.

Moreover, refining diagnostic criteria and developing targeted therapies for children with concurrent liver and kidney diseases will be essential to improve treatment outcomes. Integrated care models and longitudinal studies are needed to evaluate the long-term effects of these diseases and assess the impact of early interventions.

Future efforts in both research and clinical practice should prioritize understanding disease mechanisms, improving early detection, and creating personalized therapies. Focusing on prevention, early diagnosis, and tailored treatments will be key to enhancing long-term health outcomes for affected children.

## Figures and Tables

**Table 1:** Main features of pediatric evidence linking MASLD to KD.

Reference	Study Design	Population and Methods	Main Findings
[43]	Multicenter cohort study	1164 children (mean age 13 ± 3 years) with biopsy-confirmed NAFLD/MASLD were prospectively enrolled. Genotyping for *PNPLA3* gene polymorphism. Hyperfiltration was defined as cGFR > 135 mL/min/1.73 m^2^, while CKD stage 2 or higher as cGFR < 90 mL/min/1.73 m^2^. KD progression was defined as transition from normal to hyperfiltration or to CKD stage ≥ 2 or change in CKD by ≥1 stage.	Median cGFR of the study population was 121 mL/min/1.73 m^2^. A cGFR < 90 mL/min/1.73 m^2^ was found in 12% of patients, mostly with stage 2 CKD, while 27% showed hyperfiltration. KD was found in 39% of patients. Baseline kidney function did not correlate with the progression of liver disease over a 2-year period (*n* = 145). Hyperfiltration was independently associated with advanced liver fibrosis (OR 1.45). KD worsened in 19% of cases over the 2-year period, independent of other clinical risk factors, with a higher likelihood of progression observed in males. No association of progression of KD impairment with change in liver disease severity was observed. No significant differences in cardiometabolic comorbidities or *PNPLA3* gene polymorphism were found.
[42]	Retrospective cohort study	1037 children and adolescents (mean age 10.57 ± 2.96 years) with BMI > 95th percentile and normal kidney function (eGFR > 90 mL/min/1.73 m^2^) were enrolled. Kidney function was assessed using the Schwarz formula, normalized to body surface area. Genotyping for the *PNPLA3* I148M allele. Participants were categorized into three groups based on metabolic status: Group 1: obesity without hepatic steatosis, Group 2: obesity with hepatic steatosis (one MASLD criterion), and Group 3: obesity, hepatic steatosis, and metabolic dysregulation (≥1 MASLD criterion).	Group 3 exhibited higher ALT and HOMA-IR levels (both *p* < 0.0001) compared to other groups, with significant increases in ALT and decreases in eGFR from Group 1 to Group 3. Group 3 also had a higher frequency of the I148M allele compared to other groups (*p* < 0.0001). Carriers in group 3 showed higher diastolic BP-SDS, ALT, AST, total cholesterol, LDL, and glycemia, and lower eGFR (all *p* < 0.05). An inverse association between eGFR and both MASLD and *PNPLA3* genotypes was found (*p* = 0.011 and *p* = 0.02). This association was confirmed also in patients carrying the I148M allele (*p* = 0.006).
[131]	Cohort study	396 children and adolescents with obesity (BMI > 95th percentile) were examined. Hepatic steatosis was detected by ultrasound. Patients were stratified according to NAFLD/MASLD presence. KD was defined by eGFR < 90 mL/min/1.73 m^2^ and/or albuminuria (ACR > 30 mg/g). eGFR was calculated using the Schwartz equation, normalized to body surface area.	Children with MASLD had higher systolic and diastolic BP-SDS compared to those with NAFLD (both *p* < 0.0001). HOMA-IR and uric acid levels were significantly higher in MASLD than NAFLD group (*p* < 0.0001 and *p* = 0.006, respectively). The TG/HDL-c ratio was also higher in MASLD than NAFLD group (2.51 ± 1.51 vs. 1.89 ± 1.08, *p* < 0.0001). Children with MASLD had reduced eGFR and higher ACR values than those with NAFLD (*p* = 0.005 and *p* = 0.001, respectively). KD was more prevalent in MASLD than NAFLD (44.4% vs. 29.2%, *p* = 0.002). The adjusted OR for KD was 3.03 for MASLD (95% CI 1.59–5.77, *p* = 0.001) and 1.51 for NAFLD (95% CI 1.01–2.30, *p* = 0.05).

Abbreviations: ACR—Albumin-to-Creatinine Ratio; ALT—Alanine Aminotransferase; AST—Aspartate Aminotransferase; BMI: Body Mass Index BP: Blood Pressure; cGFR—Creatinine-Adjusted Glomerular Filtration Rate; CI—Confidence Interval; CKD—Chronic Kidney Disease; eGFR—Estimated Glomerular Filtration Rate; HOMA-IR—Homeostasis Model Assessment of Insulin Resistance; KD—Kidney Damage; LDL: Low-density lipoprotein; MASLD—Metabolic Dysfunction-Associated Steatotic Liver Disease; NAFLD—Non-Alcoholic Fatty Liver Disease; OR—Odds Ratio; PNPLA3—Patatin-Like Phospholipase Domain-Containing 3; TG/HDL-c—Triglyceride to High-Density Lipoprotein Cholesterol Ratio.

## Data Availability

The original contributions presented in the paper are included in the review article. Further inquiries can be directed to the corresponding author.

## Abbreviations

The following abbreviations are used in this manuscript:

CKD	Chronic kidney disease
HR	Hepatorenal syndrome
IR	Insulin resistance
KD	Kidney damage
MASLD	Metabolic Dysfunction-Associated Steatotic Liver Disease
MetS	Metabolic syndrome
NAFLD	Non-alcoholic fatty liver disease
OR	Odds ratio
